# Mechanistic insights into the slow peptide bond formation with D-amino acids in the ribosomal active site

**DOI:** 10.1093/nar/gky1211

**Published:** 2018-12-06

**Authors:** Sergey V Melnikov, Nelli F Khabibullina, Elisabeth Mairhofer, Oscar Vargas-Rodriguez, Noah M Reynolds, Ronald Micura, Dieter Söll, Yury S Polikanov

**Affiliations:** 1 Department of Molecular Biophysics and Biochemistry, Yale University, New Haven, CT 06520, USA; 2 Department of Biological Sciences, University of Illinois at Chicago, Chicago, IL 60607, USA; 3 Institute of Organic Chemistry at Leopold Franzens University, A-6020 Innsbruck, Austria; 4 Department of Chemistry, Yale University, New Haven, CT 06520, USA; 5 Department of Medicinal Chemistry and Pharmacognosy, University of Illinois at Chicago, Chicago, IL 60607, USA

## Abstract

During protein synthesis, ribosomes discriminate chirality of amino acids and prevent incorporation of D-amino acids into nascent proteins by slowing down the rate of peptide bond formation. Despite this phenomenon being known for nearly forty years, no structures have ever been reported that would explain the poor reactivity of D-amino acids. Here we report a 3.7Å-resolution crystal structure of a bacterial ribosome in complex with a D-aminoacyl-tRNA analog bound to the A site. Although at this resolution we could not observe individual chemical groups, we could unambiguously define the positions of the D-amino acid side chain and the amino group based on chemical restraints. The structure reveals that similarly to L-amino acids, the D-amino acid binds the ribosome by inserting its side chain into the ribosomal A-site cleft. This binding mode does not allow optimal nucleophilic attack of the peptidyl-tRNA by the reactive α-amino group of a D-amino acid. Also, our structure suggests that the D-amino acid cannot participate in hydrogen-bonding with the P-site tRNA that is required for the efficient proton transfer during peptide bond formation. Overall, our work provides the first mechanistic insight into the ancient mechanism that helps living cells ensure the stereochemistry of protein synthesis.

## INTRODUCTION

All living cells, from bacteria to human, contain both L- and D-amino acids. However, only L-amino acids are utilized for protein synthesis. The mechanism of this exclusive use of L-amino acids is not yet fully understood. This is especially notable in bacteria whose cytosol contains about a dozen different D-amino acids that are used as a carbon source, signaling molecules, or building blocks for peptidoglycan cell wall synthesis (1,2). In some bacteria, D-amino acids are present in millimolar concentrations, sometimes with the levels of D-isomers exceeding those of their L-isomers (as in the case of D-alanine and D-glutamate) (2,3). In eukaryotes, nano- to micromolar concentrations of D-amino acids are typically present in animals, plants, and fungi (4,5). Thus, organisms, from bacteria to higher eukaryotes, utilize only the L-amino acids for protein synthesis despite the presence of D-amino acids in cell cytosol.

The exclusion of D-amino acids from the ribosome-dependent protein synthesis is achieved through the cooperation of at least four independent mechanisms. First, the aminoacyl-tRNA-synthetases, which select amino acids for protein synthesis, react markedly slower with D-amino acids than with L-amino acids. For instance, tyrosyl-tRNA synthetase utilizes D-tyrosine at ∼25-fold slower rate than L-tyrosine to produce tyrosyl-tRNA (6). Second, if D-aminoacyl-tRNAs are formed, they are typically deacylated by the D-aminoacyl-tRNA deacylase (DTD) (7,8). This enzyme is conserved across the three domains of life and prevents accumulation and toxicities of D-aminoacyl-tRNAs (9). Third, if a D-aminoacyl-tRNA escapes hydrolysis by DTD, it is recognized by the elongation factor EF-Tu. However, its delivery to the ribosome occurs with ∼250-fold lower yield compared to L-aminoacyl-tRNAs (10). Finally, *in vitro* studies showed that if a D-aminoacyl-tRNA binds the ribosomal A site, it reacts with a P-site substrate at about three orders of magnitude slower rate compared to L-aminoacyl-tRNAs, illustrating that D-amino acids markedly reduce the rate of the peptide-bond formation (11). If a D-amino acid is incorporated into a nascent peptide and translocated to the P site, it might cause translation arrest, suggesting that D-amino acids also interfere with the passage of the nascent peptide through the ribosomal exit tunnel (11). Thus, cells have intricate fidelity control systems that favor preferential usage of the L-isomers over the D-amino acids at every stage of protein synthesis.

In the past years, the interest to the D-amino acid recognition by the ribosome has been revitalized due to progress in the genetic code expansion of living cells (12–14). Over the past two decades, methods have been developed that allow to genetically encode >200 non-proteinogenic amino acids to enable their ribosome-dependent and site-specific incorporation into proteins *in vivo*. These amino acids include: (i) post-translationally modified residues to explore the role of modifications in protein activity; (ii) photo-crosslinking side-chains to enable detection of transient protein interactions; (iii) fluorescent groups and self-labeling tags for improved live imaging; (iv) heavy atom-containing amino acids for X-ray crystallography; (v) residues with β-amino-acid backbone to endow proteins with resistance to proteolysis and others (12–15). However, all of the amino acids that have been successfully incorporated *in vivo* comprise only the L-isomers, while genetic encoding of D-amino acids remains a challenge.

Ribosomal synthesis of proteins with D-amino acids is desired, because site-specific replacement of L-amino acid residues with their D-isomers renders corresponding peptides protease-resistant, as it was shown for hormones and other pharmacologically active polypeptides (16–23). Also, D-amino acids are present in natural proteins (introduced via post-translational isomerization), such as bacterial lantibiotics, opioid peptides from frogs, and conotoxins (24). Therefore, the ability to perform ribosomal synthesis of D-amino acid-containing proteins is required to enable the large-scale and cost-effective production of pharmacologically active proteins and peptides.

Over the past years, messenger RNA-dependent synthesis of D-amino acid-containing proteins became possible *in vitro* via engineering of different translation machinery components. For example, protein engineering allowed the creation of aminoacyl-tRNA synthetases that selectively use D-isomer of tyrosine (25,26). Also, development of engineered catalytic RNAs, flexizymes, made possible production of D-aminoacyl-tRNAs for use in cell-free protein translation systems (27). Optimization of *in vitro* translation systems allowed synthesis of detectable amounts of peptides containing up to 10 consecutive D-amino acids (28–31). Further improvements were accomplished by random mutagenesis of ribosomal RNA (rRNA) (32,33). For instance, ribosomes carrying mutations in ^2447^GAUA^2450^ nucleotides in the 23S rRNA showed markedly improved compatibility with D-amino acids. However, these ribosome mutants were less accurate and highly toxic in *Escherichia coli* preventing their use *in vivo* (33).

The rational engineering of ribosomes to enable efficient usage of D-amino acids is currently limited due to the lack of a structural basis for the poor reactivity of D-amino acids in the peptide bond formation reaction. To overcome this limitation, we determined the crystal structure of the 70S ribosome in complex with the D-aminoacyl-tRNA mimic, ^73^ACCA^76^-D-phenylalanine (ACCA-D-Phe), bound to the ribosomal A site. Our structure reveals that the D-aminoacyl-tRNA analog binds the ribosome in a similar fashion as L-aminoacyl-tRNAs with the CCA-end binding the ribosomal A site in a canonical way and with the D-amino acid side chain accommodated by the ribosomal side chain-binding pocket. However, due to a ‘mirror’ arrangement of the substituents at the Cα-atom of the D-amino acid, the reactive α-amino group of the D-aminoacyl-tRNA analog should deviate significantly from the optimal position that is required for the nucleophilic attack onto the carbonyl carbon of the P-site substrate. Thus, our study reconciles the observed poor reactivity of D-amino acids in ribosomal protein synthesis. The reported structure provides an essential framework for the future rational design of the PTC and its surroundings to improve the usage of D-amino acids by the ribosome.

## MATERIALS AND METHODS

### Synthesis of hydrolysis-resistant D-phenylalanyl-tRNA analog

**Figure 1.**
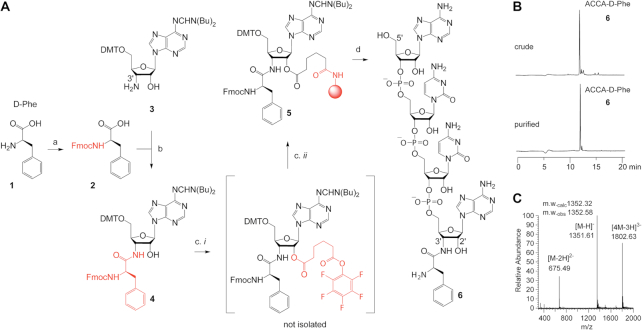
Chemical synthesis of a short hydrolysis-resistant D-phenylalanyl-tRNA analog. (**A**) Synthetic route. Letters indicate specific reaction conditions as follows: (a) 1.1 equivalent of Fmoc-Cl, Na_2_CO_3_, in 1,4-dioxane/H_2_O, room temperature, 7 h, yield 86% for compound **2**; (b) 1.3 equivalent of Fmoc-D-Phe, 1.3 equivalent of *O*-(Benzotriazole-1-yl)-*N*,*N*,*N*’,*N*’-tetramethyluronium hexafluorophosphate, 1.3 equivalent of 1-hydroxybenzotriazole hydrate, 1.5 equivalent of *N*,*N*-diisopropylethylamine in DMF, room temperature, 14 h, yield 70% for compound **4**; (c) *i*. 2.5 equivalent of adipic acid bis(pentafluorophenyl)ester, 1 equivalent of 4-(*N*,*N*-dimethylamino)pyridine in *N*,*N*-dimethylformamide/pyridine (1/1, v/v), room temperature, 1 h, yield 52% of crude ester; *ii*. ∼1 equivalent of amino-functionalized polystyrene support (GE Healthcare, Custom Primer SupportTM 200 Amino), pyridine, *N*,*N*-dimethylformamide, room temperature, one day, loading: 37 μmol/g for solid-support **5**. (d) RNA solid-phase synthesis, deprotection, and purification. DMT — 4,4′-dimethoxytrityl, Fmoc — *N*-(9- fluorenyl)methoxycarbonyl. (**B**) Anion-exchange HPLC profiles of crude (top) and purified (bottom) ACCA-D-Phe conjugate. Anion-exchange chromatography conditions: Dionex DNAPac PA-100 (4 × 250 mm) column; temperature: 60°C; flow rate: 1 ml/min; eluant A: 25 mM Tris–HCl (pH 8.0), 6 M urea; eluant B: 25 mM Tris–HCl (pH 8.0), 6 M urea, 500 mM NaClO_4_; gradient: 0–40% B in A within 25 min; UV detection at 260 nm. (**C**) LC-ESI mass spectra of the purified product ACCA-D-Phe (compound **6**).

The L-aminoacyl-tRNA mimic, cytidine-cytidine-puromycin (CC-Pmn) was obtained from Thermo Scientific. The D-aminoacyl-tRNA mimic, adenosine-cytidine-cytidine-adenosyl-D-phenylalanine (ACCA-D-Phe) was chemically synthesized. Each of these two tRNA analogs comprised a 3′-amido linkage between the 3′-terminal adenosine of the tRNA mimic and the C-terminus of the D-Phe or L-methyl-Tyr moieties to prevent hydrolysis of the analog during crystallization. The ACCA-D-Phe conjugate was produced as outlined in Figure 1A and as described below (similar to the synthesis schemes previously reported in references (34–36)). D-phenylalanine (>98% purity) was purchased from Iris Biotech GmbH and Fluka.

1. ***N*-(9-Fluorenyl)methoxycarbonyl-D-phenylalanine** (compound **2**). D-Phenylalanine **1** (0.50 g, 3.03 mmol) and Na_2_CO_3_ (1.76 g, 16.60 mmol) were suspended in 20 ml of 1,4-dioxane/H_2_O (1/1) and cooled to 0°C. At this point, 9-fluorenylmethoxycarbonyl chloride (0.86 g, 3.32 mmol) was added to the suspension and stirred for 5 min at 0°C. The ice bath was removed and the reaction mixture was stirred for 7 h at room temperature and afterwards the reaction mixture was acidified with concentrated HCl to pH 2. The resulting solution was extracted with dichloromethane (100 ml), the organic phase was washed twice with H_2_O (50 ml) and dried over Na_2_SO_4_. The solvent was evaporated and the crude product was purified by column chromatography on SiO_2_ (CH_2_Cl_2_/MeOH, 100/0–96/4 v/v). Yield: 1.01 g of *N*-(9-fluorenyl)methoxycarbonyl-D-phenylalanine **2** as white foam (86%). TLC: (CH_2_Cl_2_/MeOH, 9/1): *R*_f_ = 0.60. ESI-MS *(m/z)* [M+H]^+^ calculated 388.1543; found 388.1495.^1^H-NMR (300 MHz, DMSO-d_6_): δ = 2.81–2.93 (m, 1H, *H(a)*-C(ß, Phe)); 3.03–3.13 (m, 1H, *H(b)*-C(*ß*, Phe)); 4.15 - 4.23 (m, 4H, *H*-C(9-Fmoc), *H*-C(α, Phe), -O-C*H_2_*(Fmoc)); 7.18–7.35 (m, 8H, *H*-C(benzene) and *H*-C(fluorene); 7.41 (t, 2H, *H*-C(benzene or fluorene)); 7.65 (t, 2H, *H*-C(benzene or fluorene)); 7.43 (d, 1H, -*H*N(Phe)); 7.88 (d, 2H, *H*-C(fluorene)); 12.74 (s,1H, -O*H*) ppm. ^13^C-NMR (150 MHz, DMSO-d_6_): δ = 36.43 (C(ß, Phe)); 46.55 (C(9, Fmoc)); 55.46 (C(α, Phe)); 65.58 (C(methylene, Fmoc)); 120.05, 125.19, 125.26, 126.33, 127.02, 127.59, 128.14, 129.07, 137.96, 140.64, 143.72 (C(benzene and fluorene)); 155.90 (C=O(Fmoc)); 172.28 (C=O(Phe)) ppm.
2. ***N*^6^-[(Di-n-butylamino)methylene]-3′-[*N*-(9-fluorenyl)methoxycarbonyl-D-phenylalanyl]amino-3′-deoxy-5′-O-(4,4′-dimethoxytrityl)-D-adenosine** (compound **4**). Fmoc-protected D-phenylalanine **2** (75 mg, 0.19 mmol) was dissolved in 3 ml DMF followed by addition of *O*-(benzotriazol-1-yl)-*N,N,N',N'*-tetramethyluronium hexafluorophosphate (HBTU, 74 mg, 0.20 mmol), 1-hydroxybenzotriazole hydrate (HOBt, 30 mg, 0.20 mmol), and *N,N*-diisopropylethylamine (DIPEA, 38 μl, 0.23 mmol). After 10 min of activation, 3′-amino-*N*^6^-[(di-*n*-butylamino)methylene]-3′-deoxy-5′-*O*-(4,4′-dimethoxytrityl)-D-adenosine **3** (35) (106 mg, 0.15 mmol, in 1 ml DMF) was added and the mixture was stirred for 14 h overnight at room temperature. Then, the solvent was evaporated, the residue dissolved in CH_2_Cl_2_ and washed consecutively with half-saturated aqueous NaHCO_3_ solution, 5% citric acid solution, and saturated NaCl solution. The organic layer was dried (Na_2_SO**_4_**) and evaporated, and the crude product was purified via SiO_2_ column chromatography using a gradient from 1 to 5% methanol in dichloromethane. Yield: 112 mg of compound **4** as white foam (70%). TLC ((CH_2_Cl_2_/MeOH, 92/8). *R*_f_ = 0.37. ^1^H NMR (700 MHz, CDCl_3_) δ = 0.96 (q, 6H, N(CH_2_CH_2_CH_2_C*H*_3_)_2_; 1.36–1.44 (m, 4H, N(CH_2_CH_2_C*H_2_*CH_3_)_2_); 1.64–1.71 (m, 4H, N(CH_2_C*H_2_*CH_2_CH_3_)_2_); 3.02–3.09 (m, 2H, *H(a)*-C(ß, Phe) and *H(b)*-C(ß, Phe); 3.38–3.44 (m, 3H, N(C*H_2_*CH_2_CH_2_CH_3_) and *H(a)-*C(5′)); 3.50 (d, 1H, *H(b)*-C(5′)); 3.68–3.78 (m, 8H, N(C*H_2_*CH_2_CH_2_CH_3_) and 2xOC*H_3_*(DMT)); 4.21 (m, 1H, *H*C(9, Fmoc)); 4.30 (m, 1H, *H*-C(4′)); 4.35–4.46 (m, 3H, *H*-C(α, Phe) and OC*H_2_*(Fmoc)); 4.53 (q, 1H, H-C(3′)); 4.70 (m, 1H, *H*-C(2′)); 5.31 (m, 1H, *H*N(Phe)); 5.37 (s, br, 1H, *H*-C(1′)); 5.89 (s, br, 1H, *H*O-C(2′)); 6.55 (s, br, 1H, *H*N-C(3′)); 6.78 (d, 4H, *H-*C(ar)); 7.15–7.42 (m, 18H, *H*-C(ar)); 7.51–7.58 (m, 2H, *H*-C(ar)); 7.77 (d, 2H, *H*-C(ar)); 8.14 (s, 1H, *H*-C(8)); 8.49 (s, 1H, *H*-C(2)); 9.06 (s, 1H, *H*C=N-C(6)) ppm). ^13^C NMR (175 MHz, CDCl_3_) δ = 13.70, 13.94 (N(CH_2_CH_2_CH_2_*C*H_3_)_2_); 19.78, 20.21 (N(CH_2_CH_2_*C*H_2_CH3)_2_); 29.72, 30.98 (N(CH_2_CH_2_CH_2_CH_3_)_2_); 38.71 ((C(ß), Phe)); 45.24 N(CH_2_CH_2_CH_2_CH_3_)_2_); 47.12 (C(9, Fmoc); 51.95 N(CH_2_CH_2_CH_2_CH_3_); 52.44 (C(3′)); 56.35 (2 × O-CH_3_(DMT)); 56.57 (C(α,Phe)) 63.34 (C(5′)); 67.10 (C(methylene, Fmoc)); 74.69 ((C(2′)); 83.83 (C(4)); 86.56; 91.21 (C(1)); 113.26; 120.12; 125.14; 126.49; 126.96; 127.21; 127.34; 127.88; 127.95; 128.32; 128.96; 129.39; 130.20; 135.73; 135.75; 136.34; 139.57 (C(8)); 141.43; 143.75; 143.89; 144.50; 150.67, 152.41; 155.97; 158.60; 158.60; 158.89; 160.53; 171.36 (C=O (Phe)) ppm. ESI-MS (*m/z*): [M+H]^+^ calculated for C_64_H_69_N_8_O_8_, 1077.5233; found 1077.5247.
3. **DMTO-rA3′-NH-D-Phe-NHFmoc solid support** (compound **5**). To a solution of compound **4** (80 mg, 0.074 mmol) in DMF and pyridine (1.0 ml each) was added DMAP (9 mg, 0.074 mmol) and bis(pentafluorophenyl) adipate (88.7 mg, 0.19 mmol). The mixture was stirred for one hour followed by evaporation of the solvents. The residue was applied to filtration over SiO_2_ yielding 53 mg of the crude ester as a white foam (TLC (20% acetone in CH_2_Cl_2_) *R*_f_ = 0.51). The crude ester (53 mg, 0.039 mmol) was dissolved in dry DMF (2.0 ml), and pyridine (80 μl) was added. To this solution, amino-functionalized support (GE Healthcare, Custom Primer Support™ 200 Amino, 160 mg) was added, and the suspension was agitated for 18 h at room temperature. Subsequently, the beads were collected on a Büchner funnel and washed with DMF, methanol, and CH_2_Cl_2_. For capping of unreacted amino groups, the beads were treated with a mixture of solution Cap A (0.2 M phenoxy acetic anhydride in THF, 10 ml) and solution Cap B (0.2 M *N*-methyl imidazole, 0.2 M sym-collidine in THF, 10 ml) and agitated for 10 min at room temperature. The suspension was filtrated again; the beads were washed with acetonitrile, methanol, and CH_2_Cl_2_ and dried under vacuum. Loading of the support **5** was 37 μmol/g.
4. **RNA synthesis** The ACCA moiety was assembled on an ABI 392 Nucleic Acid Synthesizer following standard synthesis protocols using solid support **5**. Detritylation (120 s): dichloroacetic acid/1,2-dichloroethane (4/96); coupling (120 s): phosphoramidites (0.1 M in acetonitrile, 130 ml) were activated with benzylthiotetrazole (0.3 M in acetonitrile, 180 μl); capping (2 × 10 s, Cap A/Cap B = 1/1): Cap A: phenoxyacetic anhydride (0.2 M in THF), Cap B: N-methyl imidazole (0.2 M), sym-collidine (0.2 M) in THF; oxidation (20 s): I_2_ (0.2 M) in THF/pyridine/H_2_O (35/10/5). Amidites, benzylthiotetrazole, and capping solutions were dried over activated molecular sieves (3 Å) overnight.
5. **Deprotection of the 3′-D-phenylalanyl-ACCA conjugate**. The deprotection and cleavage of the conjugate from the solid support proceeded in three steps. 1. Fmoc deprotection. In the ABI synthesis column, the solid support was treated with a solution of 20% piperidine in acetonitrile (10 ml, 10 min), washed with acetonitrile, and dried. 2. Acyl deprotection and cleavage from the solid support. For the conjugates synthesized on solid support **5**, the beads were transferred into a screw-capped Eppendorf tube, and equal volumes of 28% aqueous ammonia (0.5 ml) and methylamine in H_2_O (40%, 0.5 ml) were added. After 4-hour shaking at room temperature, the supernatant was filtered and evaporated to dryness. 3. 2′-*O*-TOM deprotection. The obtained residue was treated with TBAF·3 H_2_O in THF (1 M, 1 ml) overnight at room temperature. The reaction was quenched by the addition of triethylammonium acetate (TEAA) (1 M, pH 7.4, 1 ml). After reducing the volume of the solution, it was applied on a size-exclusion chromatography column (GE Healthcare, HiPre 26/10 desalting, 2.6 × 10 cm, Sephadex G25). By eluating with H_2_O, the conjugate-containing fractions were collected and evaporated to dryness, and the residue was dissolved in H_2_O (1 ml). Analysis of the crude products was performed by anion-exchange chromatography on a Dionex DNAPac PA-100 column (4 × 250 mm) at 60°C. Flow rate: 1 ml/min; eluent A: 25 mm Tris–HCl (pH 8.0), 6 M urea; eluent B: 25 mM Tris–HCl (pH 8.0), 0.5 M NaClO_4_, 6 M urea; gradient: 0–60% B in A within 45 min or 0–40% B in A within 30 min, UV detection at λ = 260 nm.
6. **Purification of the 3′-D-phenylalanyl-ACCA conjugate**. The crude conjugate was purified on a semipreparative Dionex DNAPac PA-100 column (9 × 250 mm) at 60°C with a flow rate of 2 ml/min (for eluents, see above). Fractions containing the conjugate were loaded on a C18 SepPak Plus cartridge (Waters/Millipore), washed with 0.1–0.15 M (Et_3_NH)^+^HCO_3_^−^, H_2_O, and eluted with H_2_O/CH_3_CN (1:1). Conjugate-containing fractions were evaporated to dryness and dissolved in H_2_O (1 ml). The quality of the purified conjugate was analyzed by analytical anion-exchange chromatography (Figure 1B). The molecular weight of the synthesized conjugate was confirmed by LC-ESI mass spectrometry (Figure 1C). Yields were determined by UV photometric analysis of conjugate solutions. The final compound was dissolved in water to achieve ∼50 mM concentration for stock solutions and later used in co-crystallization experiments.

### Crystallographic structure determination

Ribosome–mRNA–tRNA complex was pre-formed by programming 5 μM 70S *Tth* ribosomes with 10 μM mRNA and incubation at 55°C for 10 min, followed by addition of 20 μM P-site (tRNA_i_^Met^) and 100 μM A-site (CC-Pmn or ACCA-D-Phe) substrates (with minor changes from (37)). Each of these two steps was allowed to reach equilibrium for 10 min at 37°C in the buffer containing 5 mM HEPES–KOH (pH 7.6), 50 mM KCl, 10 mM NH_4_Cl, and 10 mM Mg(CH_3_COO)_2_, Crystals were grown by vapor diffusion in sitting drop crystallization trays at 19°C. Initial crystalline needles were obtained by screening around previously published ribosome crystallization conditions (38–40). The best-diffracting crystals were obtained by mixing 2–3 μl of the ribosome complexes with 3–4 μl of a reservoir solution containing 100 mM Tris–HCl (pH 7.6), 2.9% (w/v) PEG-20K, 7–12% (v/v) MPD, 100–200 mM arginine, 0.5 mM β-mercaptoethanol (41). Crystals appeared within 3–4 days and grew up to 150 × 150 × 1600 μm in size within 10–12 days. Crystals were cryo-protected stepwise using a series of buffers with increasing MPD concentrations until reaching the final concentration of 40% (v/v) MPD, in which they were incubated overnight at 19°C. In addition to MPD, all stabilization buffers contained 100 mM Tris–HCl (pH 7.6), 2.9% (w/v) PEG-20K, 50 mM KCl, 10 mM NH_4_Cl, 10 mM Mg(CH_3_COO)_2_ and 6 mM β-mercaptoethanol. CC-Pmn or ACCA-D-Phe were not added to any of the cryo-protection solutions. After stabilization, crystals were harvested and flash frozen in a nitrogen cryo-stream at 80°K (Oxford Cryosystems).

Diffraction data were collected at the beamlines 24ID-C and 24ID-E at the Advanced Photon Source (Argonne National Laboratory, Argonne, IL). A complete dataset for each ribosome complex was collected using 0.979 Å wavelength at 100K from multiple regions of the same crystal using 0.3° oscillations. The raw data were integrated and scaled using the XDS software package (42). All crystals belonged to the primitive orthorhombic space group *P*2_1_2_1_2_1_ with approximate unit cell dimensions of 210 Å × 450 Å × 620 Å and contained two copies of the 70S ribosome per asymmetric unit. Each structure was solved by molecular replacement using PHASER from the CCP4 program suite (43). The search model was generated from the previously published structure of the *T. thermophilus* 70S ribosome with all modifications and with bound mRNA and P-site tRNA (PDB entry 4Y4P from (41)). The initial molecular replacement solutions were refined by rigid body refinement with the ribosome split into multiple domains, followed by 10 cycles of positional and individual B-factor refinement using PHENIX (44). Non-crystallographic symmetry restraints were applied to 4 domains of the 30S ribosomal subunit (head, body, spur, helix 44), and four domains of the 50S subunit (body, L1-stalk, L10-stalk, C-terminus of the L9 protein).

**Figure 2.**
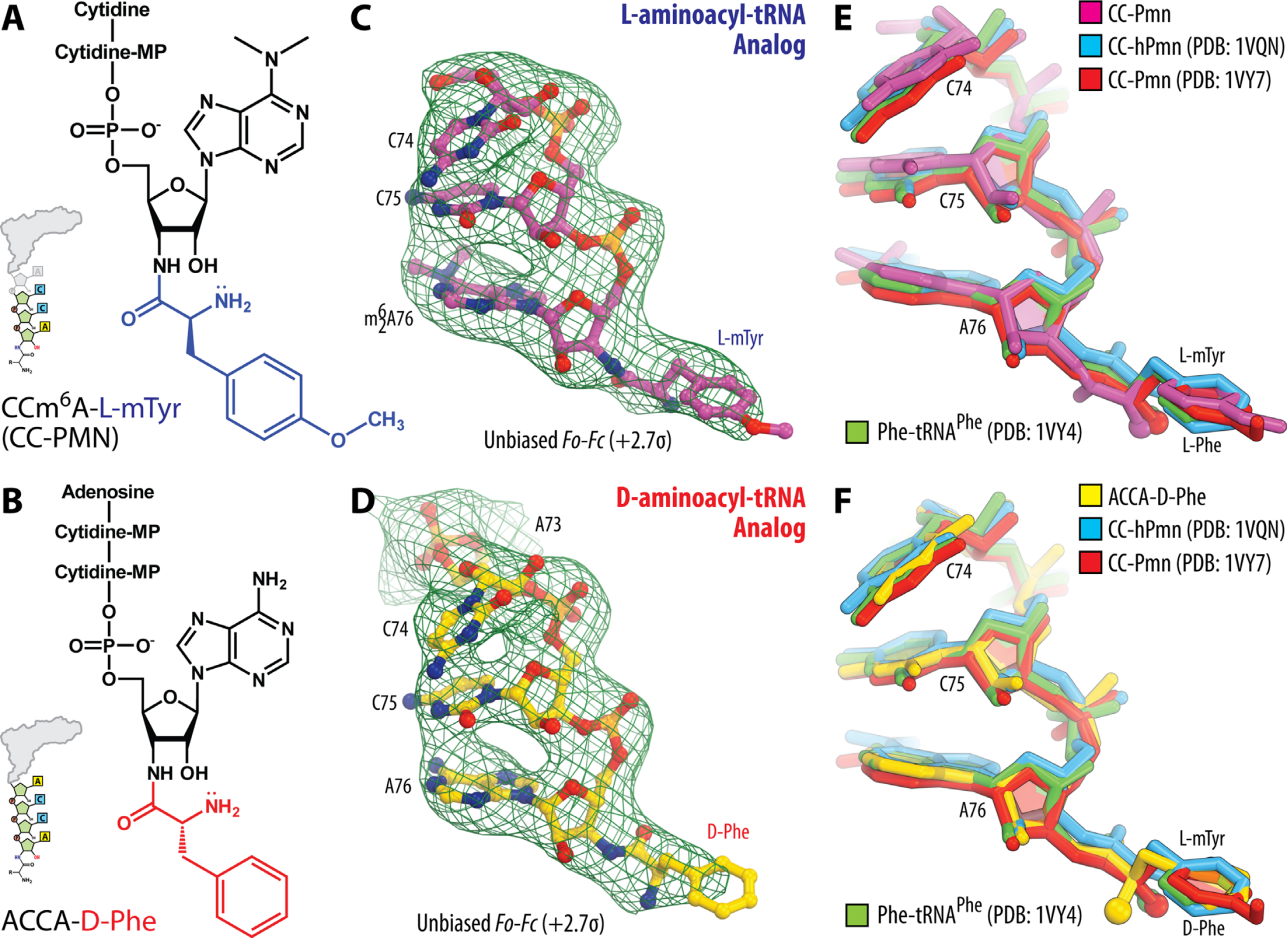
The electron density maps allow to unambiguously position L- and D-amino acid side chains bound to the ribosomal active site. (**A, B**) Chemical structures of the L-aminoacyl-tRNA mimic CC-Pmn (A), and of the D-aminoacyl-tRNA mimic ACCA-D-Phe (B). The amino acid moieties of L-methyl-tyrosine and D-phenylalanine are highlighted in blue and red, respectively. (**C, D**) Unbiased *F_o_-F_c_* electron difference Fourier maps of CC-Pmn (C), and ACCA-D-Phe (D). The refined model of each compound is displayed in its respective electron density map before the refinement (green mesh). Carbon atoms are colored yellow for ACCA-D-Phe, and magenta for CC-Pmn. Nitrogens are colored blue; oxygens are red; phosphorus atoms are orange. Each of the difference electron density maps is contoured at 2.7σ. Note that, due to the presence of an additional 5′-terminal adenine nucleotide in the ACCA-D-Phe in comparison to the CC-Pmn, each of these compounds can be unambiguously distinguished in the electron density maps. (**E, F**) Comparison of the current structures with the previously reported structures of the A-site-bound short and full-length tRNA substrates. Shown are superimposed ribosome-bound (E) CC-Pmn (magenta, current model) or (F) ACCA-D-Phe (yellow, current model) and CC-hPmn (blue, PDB entry 1VQN (48)), CC-Pmn (red, PDB entry 1VY7 (37)), and Phe-NH-tRNA^Phe^(green, PDB entry 1VY4 (37)). All structures were aligned based on domain V of the 23S rRNA. Note that differences between the compared structures of the A-site substrates are within experimental error.

Atomic models of CC-Pmn and ACCA-D-Phe were generated from their known chemical structures using PRODRG online software (45), which was also used to generate restraints for energy minimization and refinement based on idealized 3D geometry. Atomic models and restraints were used to fit/refine each of the tRNA mimics into the obtained unbiased electron density maps (Figure 2). The final model of the 70S ribosome in complex with CC-Pmn or ACCA-D-Phe and mRNA/tRNAs was generated by multiple rounds of model building in COOT (46), followed by refinement in PHENIX (44). The statistics of data collection and refinement are compiled in Table 1. All figures showing atomic models were generated using PyMol software (www.pymol.org).

**Table 1.** X-ray data collection and refinement statistics

	70S complex with P-site tRNA and CC-Pmn	70S complex with P-site tRNA and ACCA-D-Phe
***Diffraction*** ***data***		
Space group	*P*2_1_2_1_2_1_	*P*2_1_2_1_2_1_
Unit cell dimensions, Å (*a* × *b* × *c*)	212.24 × 452.84 × 620.30	211.39 × 452.15 × 617.64
Wavelength, Å	0.9795	0.9795
Resolution range (outer shell), Å	213–3.70 (3.80–3.70)	255–3.70 (3.80–3.70)
I/σI (outer shell with I/σI = 1)	6.87 (0.88)	5.59 (0.86)
Resolution at which I/σI = 1, Å	3.70	3.70
Resolution at which I/σI = 2, Å	4.05	4.10
CC(1/2) at which I/σI = 1, %	18.8	24.9
CC(1/2) at which I/σI = 2, %	49.0	65.0
Completeness (outer shell), %	99.4 (99.0)	98.5 (98.5)
*R*_merge_ (outer shell)%	23.7 (250.8)	17.7 (143.7)
No. of crystals used	2	1
No. of Reflections Used:		
Observed	5 428 817	2 154 810
Unique	627 226	615 531
Redundancy (outer shell)	8.65 (7.95)	3.50 (3.14)
Wilson *B*-factor, Å^2^	135.2	123.6
***Refinement***		
R_work_/R_free_, %	23.7/27.8	23.6/27.9
*No. of non-hydrogen atoms*		
RNA	194 357	194 333
Protein	90 976	90 976
Ions (Mg, K, Zn, Fe)	1 184	1 367
Waters	125	247
*Ramachandran plot*		
Favored regions, %	94.15	94.46
Allowed regions, %	5.21	4.90
Outliers, %	0.64	0.64
*Deviations from ideal values (RMSD)*		
Bond, Å		
	0.003	0.003
Angle, degrees	0.635	0.635
Chirality	0.034	0.034
Planarity	0.004	0.004
Dihedral, degrees	13.650	13.625
Average B-factor (overall), Å^2^	113.1	103.8

*R*_merge_ = Σ |*I* – <*I*>| / Σ *I*, where I is the observed intensity and <*I*> is the average intensity from multiple measurements.

*R*_work_ = Σ|*F*_obs_ – *F*_calc_| / Σ*F*_obs_. For calculation of *R*_free_, 5% of the truncated dataset was excluded from the refinement.

## RESULTS

### Crystal structures of the 70S ribosome in complex with L- and D-aminoacyl-tRNA analogs in the ribosomal A site

To provide structural insights into the poor reactivity of the D-aminoacyl-tRNAs in the peptide bond formation we determined the crystal structure of *T. thermophilus* 70S ribosomes in complex with hydrolysis-resistant analogs of aminoacyl-tRNAs. We used cytidyl-cytidyl-puromycin (CC-Pmn) as an L-aminoacyl-tRNA analog that carried L-methyl-tyrosine residue (Figure 2A, L-mTyr) and adenyl-cytidyl-cytidyl-adenylyl-D-phenylalanine (ACCA-D-Phe) as a D-aminoacyl-tRNA analog that carried D-phenylalanine residue (Figure 2B, D-Phe). These short analogs mimic the 3-terminal CCA-ends of the acceptor stem of full-length tRNAs. Both analogs carried a D- or L-amino acid attached to the 3′-terminal nucleotide via the amide linkage (instead of the naturally occurring ester bond) to prevent spontaneous hydrolytic deacylation during the crystallization (Figure 2A, B). Using either of these compounds as an A-site substrate, we determined their crystal structures in complex with the *T. thermophilus* 70S ribosomes carrying messenger RNA and tRNA_i_^fMet^ in the P site (Materials and Methods). Although the P-site tRNA in both of our complexes was represented by the deacylated tRNA_i_^fMet^, which is not strictly physiological, previous studies have shown that aminoacylation status of the P-site tRNA does not affect conformation of the amino acid attached to the A-site tRNA substrate (37,47–49). Therefore, it is reasonable to assume that the conformation and interactions of the A-site substrates in our structures are identical to those seen in physiologically more relevant complexes of the ribosome.

Both crystal structures were determined at 3.7 Å resolution by molecular replacement using the atomic coordinates of the *T. thermophilus* 70S ribosome with the A-site tRNA removed (PDB entry 4Y4P (41)) (Table 1). The unbiased difference Fourier maps revealed unique positive density peaks carrying characteristic features of the CC-Pmn and ACCA-D-Phe analogs bound to the ribosomal A site (Figure 2C, D). To build the structural models of the A-site substrates, we used the best-fit placement of ACCA-D-Phe and CC-Pmn molecules into the electron density maps and subsequent crystallographic refinement (Materials and Methods). Because the resolution of our datasets did not allow direct visualization of the individual chemical groups, the accurate model building was aided by the chemical restraints. Here, we need to note that the ribosome structure in complex with A-site CC-Pmn has been reported previously at ∼1Å-higher resolution (37). The only reason why we determined it again is to validate the accuracy of our structural models determined at 3.7Å resolution.

We next asked if our maps provide a sufficient level of detail to gain mechanistic insights into ribosome stereoselectivity. For this purpose, we compared our 3.7 Å-resolution structure of the 70S/CC-Pmn-complex with similar or identical structures that were determined previously at 2.4–2.8 Å resolution (Figure 2E) (37,48). Our comparison revealed no significant differences in the location and orientation of the CC-Pmn molecule on the 70S ribosome between the new and the previous structures (Figure 2E). In all analyzed structures, the L-mTyr was tightly fit into the A-site pocket due to shape complementarity between the ribosomal A-site and the amino acid backbone. This similarity of L-mTyr conformation in different crystal structures illustrated that, despite limited detail, our 3.7Å-resolution maps allowed reliable model building, which was due to (i) the strong signal from bulky aromatic side chains of amino acids, (ii) chemical restraints used during real-space fitting and model refinement and (iii) limited volume of the A site pocket that leaves A-site substrates little freedom to move.

### CCA-end of the D-aminoacyl-tRNA manifests canonical interactions with the ribosome

**Figure 3.**
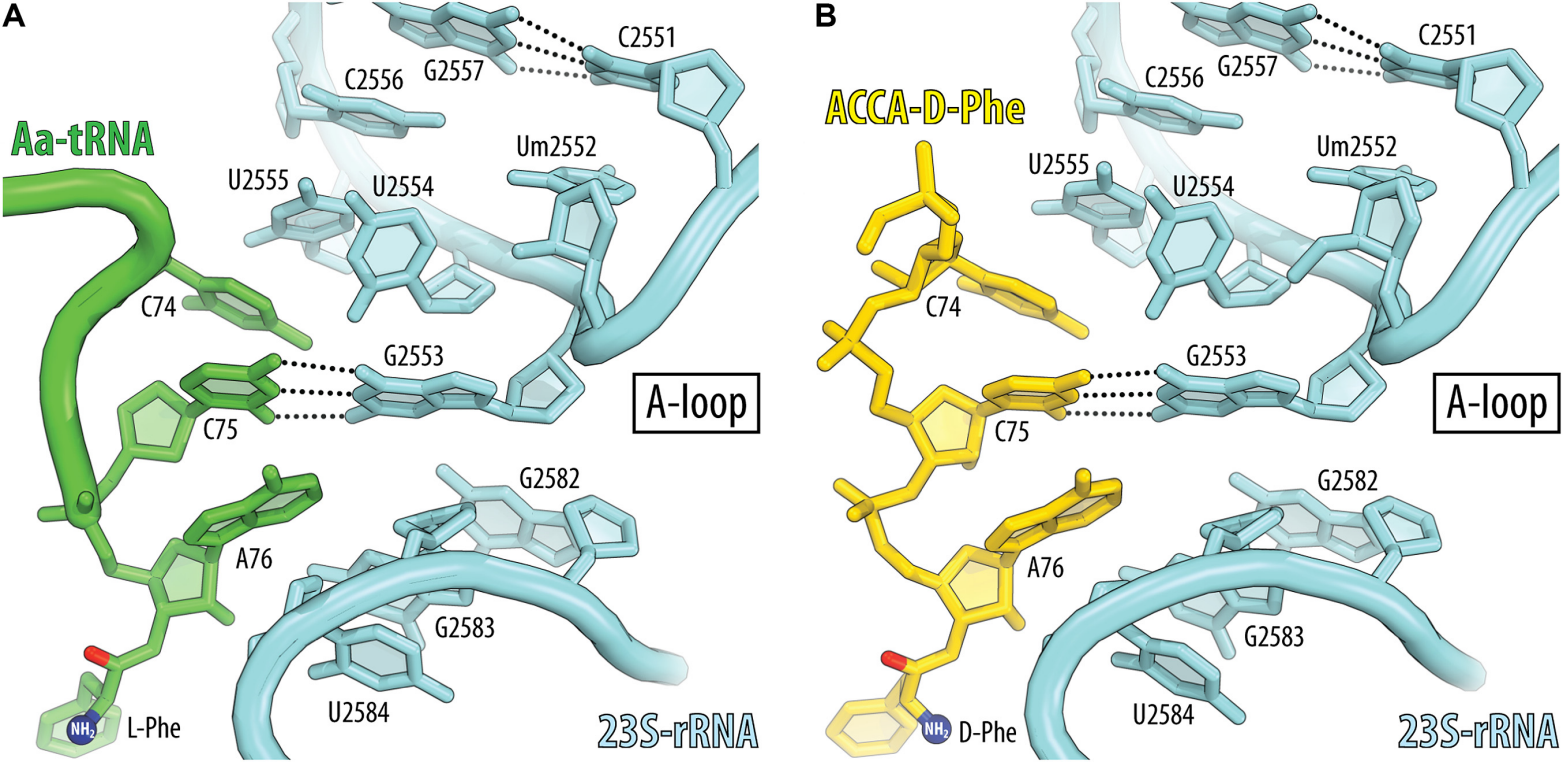
D-aminoacyl-tRNA analog establishes canonical A-loop interactions. Watson-Crick base-pairing between the penultimate cytidine of the (**A**) L-Phe-tRNA^Phe^ (green, PDB entry 1VY4 (37)) or (**B**) D-aminoacyl-tRNA analog ACCA-D-Phe (yellow) and the nucleotide G2553 in the A-loop (Helix 92) of the 23S rRNA (light blue). Note that these A-loop interactions play a key functional role in accommodation and proper positioning of the substrate in the A site of the ribosome.

Next, we assessed whether the observed poor reactivity of D-aminoacyl-tRNAs might be caused by suboptimal positioning of the CCA-end in the A site. In the case of canonical L-aminoacyl-tRNAs, their binding to the A site results in specific interactions of the CCA-end with the A-loop (Helix 92) of the 23S rRNA, in which the tRNA residue C75 forms Watson-Crick base-pair with the nucleotide G2553 (here and throughout the text we use *E. coli* numbering of rRNA nucleotides) (Figure 3A) (47). This interaction is required for the proper positioning of the aminoacyl-tRNA substrate in the PTC (47). Our electron density maps revealed that, in both crystal structures, the CCA-ends of aminoacyl-tRNA analogs establish canonical contacts with the A-loop (Figure 3B), illustrating that the presence of D amino acid residue does not impede recognition of the CCA-end by the ribosome (Figure 2E, F).

### Side chains of L- and D-amino acid residues bind the A site in a similar fashion

**Figure 4.**
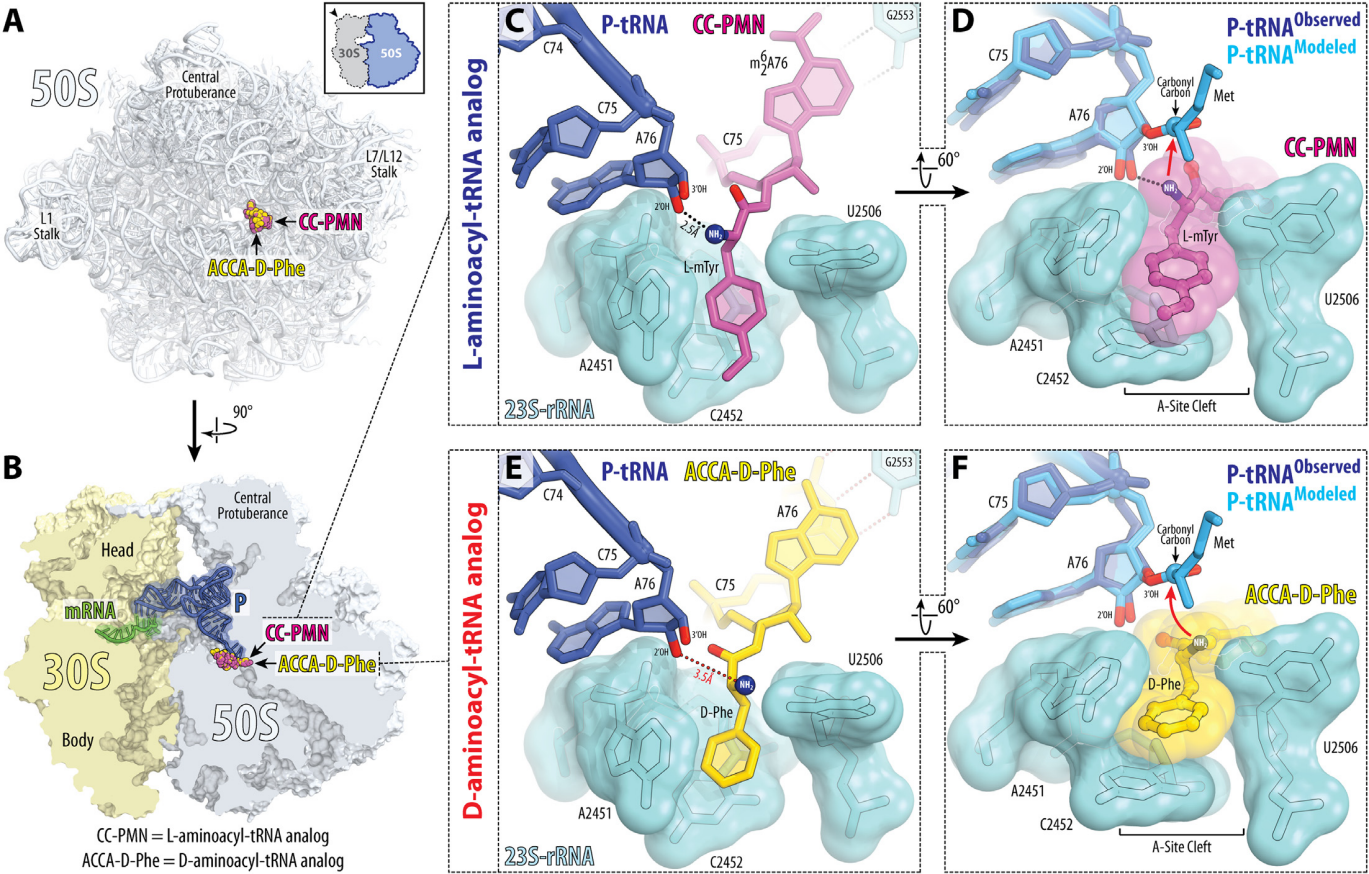
Side chains of both L- and D-amino acids occupy the A-site cleft of the ribosome. (**A, B**) Overview of the CC-Pmn (magenta) and ACCA-D-Phe (yellow) binding sites in the *T. thermophilus* 70S ribosome viewed from the PTC down the tunnel as indicated by the inset (A), or as a cross-cut section through the ribosome (B). The 30S subunit is shown in light yellow, the 50S subunit is in light blue, the mRNA is in green, and the P-site tRNA is in dark blue. (**C–F**) Close-up views of the CC-Pmn (C, D) and ACCA-D-Phe (E, F) bound in the A-site cleft of the PTC. The *E. coli* nucleotide numbering is used throughout. In (C, D), H-bond between the α-amino group and the 2′-OH of the A76 of the P-site tRNA is shown with the black dotted line. This H-bond is pivotal to optimally orient α-amine for an in-line nucleophilic attack onto the carbonyl carbon of the P-site substrate (red arrow). Note that the formation of the same H-bond is not plausible for ACCA-D-Phe because its α-amino group is located further away and oriented towards the nucleotide U2506 (red dotted line). The ability of this group to attack the P-site substrate from this remote location is expected to be reduced due to the non-optimal geometry (curved red arrow). In (D, F), the aromatic side chains of the CC-Pmn and ACCA-D-Phe are highlighted by semi-transparent spheres to illustrate their tight binding in the A-site cleft. Also in (D, F), the observed deacylated P-site tRNA_i_^Met^ (dark blue) is superimposed with the aminoacylated fMet-tRNA_i_^Met^ (light blue, PDB entry 1VY4 (37)) based on alignment of the 23S rRNA. Note that the superimposed tRNAs structures are nearly identical even though one is determined at 3.7Å (observed) and the other – at 2.55Å (modeled).

We next asked how D-amino acid binding to the A site is compared to that of L-amino acids. The electron density maps revealed the backbones of both L- and D- amino acids, as well as the entire side chain of the L-methyl-tyrosine (Figures 2C and 4C, D). The side chain of the D-phenylalanine (Figures 2D and 4E, F) was also visible in the electron density map, although only up to the Cγ atom, pointing to the partial flexibility of the D-amino acid side chain. The position of the Cγ atom of the D-phenylalanine side chain and the fact that the bulky side chain of D-Phe should tightly fit into the A-site cleft indicates that the D-Phe side chain is fully accommodated into the A-site cavity because any other orientation of the tip of D-Phe side chain would result in collisions with the surrounding rRNA nucleotides (Figure 4C–F). Thus, our data indicate that not only the CCA-end of the D-aminoacyl-tRNA analog forms canonical interactions with the ribosome but also the D-amino acid side chain binds the A-site cleft in a fashion similar to that of the L-amino acids side chains before the peptide bond formation takes place.

### D-amino acid adopts a poorly reactive conformation in the ribosomal A site

**Figure 5.**
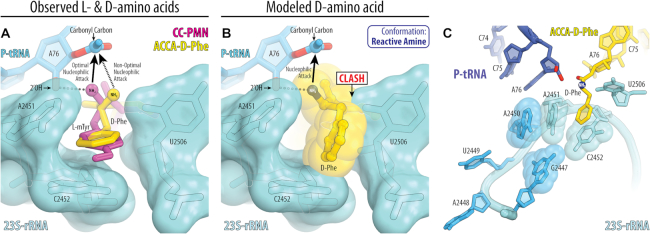
Reactive conformation of the D-amino acid is likely prevented by the conserved rRNA residues in the peptidyl-transferase center. (**A**) Comparison of the observed structures of the L- and D-aminoacyl-tRNA analogs bound to the ribosome. Shown are the 23S-rRNA-aligned energy-minimized conformations of CC-Pmn (magenta) and ACCA-D-Phe (yellow) bound to the ribosomal A site. Note that, due to the opposite chirality of the Cα-atoms, the α-amino group of the D-phenylalanine is positioned further away from the carbonyl carbon of the P-site substrate resulting in reduced reactivity. (**B**) The conformation of the D-phenylalanine, in which its α-amino group is aligned for the optimal nucleophilic attack onto the carbonyl carbon of the P-site substrate. In this state, the side chain of D-amino acid (especially Cβ-atom) severely clashes with the key functional nucleotide U2506 of the PTC. (**C**) Mutations in the 23S rRNA that improve ribosomal usage of D-amino acids. Relative locations of the 23S rRNA residues A2451 and C2452 forming the A-site cleft (light blue spheres) and the residues G2447, A2448, U2449 and A2450, whose mutations improve utilization of the D-amino acids by the ribosome (blue). Shown is the close-up view of the PTC with bound P-site tRNA (dark blue) and A-site short substrate ACCA-D-Phe (yellow). Note that residues G2447 and A2450 (blue spheres) are located near the A-site cleft. Mutations of these purine residues to smaller pyrimidines might lead to either an increased size of the A-site cleft or increased flexibility of the adjacent residues forming the A-site cleft.

Next, we checked whether binding of the D-Phe side chain to the A-site cleft affects the conformation of its reactive α-amino group. Of course, at the 3.7 Å resolution, it is impossible to identify the exact location of the α-amino group, although this is also impossible even at higher resolutions at which ribosome structures were reported previously (such as 2.3–2.5 Å). What is crucial here is the fact that we observe electron density for the part of the D-Phe side chain. And by applying ideal chemical restraints, from the known locations of the Cα, Cβ and Cγ atoms we can unequivocally deduce the location of the reactive α-amino group and compare it with that of L-mTyr (Figure 5A).

In case of L-amino acids, the α-amino group (such as the one in CC-Pmn) forms a hydrogen bond (H-bond) with the 2′-hydroxyl of the nucleotide A76 of the P-site tRNA (Figure 4C, black dotted line), which plays critical role in (i) its positioning for the optimal nucleophilic attack onto the carbonyl carbon of the P-site substrate and (ii) the subsequent shuttling of the protons (Figure 5A, solid arrowhead) (37). By contrast, in our structure of ribosome-bound ACCA-D-Phe substrate, the α-amino group of the D-amino acid is directed not towards, but away from the P-site tRNA (Figure 5A). In this orientation, the α-amino group is unlikely to form such H-bond due to the at least 3.5Å distance between the α-amino group and the 2′-OH group of the P-site tRNA and unfavorable geometry (Figures 4E and 5A). As a result, the nucleophilic attack from this position is likely to be less efficient due to the larger distance between the reactive α-amino group and the carbonyl carbon of the P-site substrate (Figure 5A, dashed arrowhead). Also, a defined path for abstraction of the proton from the attacking α-amine is not arranged (37), which might be another reason for the decreased reactivity of the D-amino acid residues during the peptide bond formation reaction.

### The poorly reactive conformation of the D-amino acid is likely caused by the specific rRNA residues in the ribosomal A site

We finally asked what prevents the α-amino group of the D-amino acid residue from adopting a reactive conformation in the A site similar to that of the L-amino acids. To answer this question, we explored the range of sterically allowed conformations of the D-phenylalanine in the ribosomal A site by using *in silico* modeling. We found that if the reactive α-amino group of the D-amino acid is positioned for the optimal nucleophilic attack onto the carbonyl carbon of the P-site substrate (37,48), then the side chain would clash with the universally conserved nucleotide U2506 in the PTC (Figure 5B). In this case, the largest collision is observed between the Cβ-atom of the D-phenylalanine and the base of U2506 suggesting that even the smallest D-amino acid (such as D-alanine) is unlikely to adopt the optimal reactive conformation of the α-amine in the PTC of the wild-type ribosome.

## DISCUSSION

Here we report the first crystal structure of the ribosome in complex with a ‘mirror’ substrate, an analog of D-aminoacyl-tRNA. This structure provides mechanistic insights into the poor reactivity of D-amino acids in the peptide bond formation and illustrates one of the mechanisms that allow cells to prevent co-translational incorporation of D-amino acids into natural proteins.

### The role of the A-site cleft in the amino acid positioning in the A site

A-site cleft binds the side chains of incoming amino acids and plays a critical role in the positioning of the incoming amino acids in the PTC. Previously, all the observed L-amino acid residues were shown to adopt a uniform position in the ribosomal A site, where the Cβ-atom (and all other atoms) of the side chain is always directed into the A-site cleft (37,47,48,50,51). Such orientation was suggested to help physically exclude amino acid side chain from the catalytic center of the ribosome and, thereby, prevent potential steric clashes and non-desired chemical reactivities of amino acid side chains in the peptide bond formation. Our observation that the D-amino acid side chain accommodates into the A-site cleft suggests that this conserved component of the ribosomal catalytic center might also be involved in the stereospecificity control of protein synthesis.

### Key determinants of L- versus D-amino acid discrimination

Previous studies suggested two alternative models of how ribosome can discriminate between the two possible chiralities of the incoming amino acids and reject D-amino acids from the use in protein synthesis. One model, based on the molecular modeling attempts using pioneering structures of archaeon *Haloarcula marismortui*, assigned the critical role in rejecting D-amino acids to the nucleotide U2585 in the 23S rRNA (52,53). In another model, based on structural analysis of the pre-attack state of the peptide bond formation reaction, the critical role in discriminating amino acid chirality has been assigned to the nucleotide U2506 (54). The main difference between these two models stemmed from the lack of knowledge about the orientation of the D-amino acid in the ribosomal A site, particularly regarding the orientation of the side chain. Our current structure, illustrating how the D-amino acid side chain binds the A-site cleft of the ribosome, is consistent with the model in which the key discriminatory role is played by the U2506 residue (Figure 5B).

### Insights into the mechanism of poor D-amino acid reactivity in the ribosomal A site

Our structure suggests that the poor reactivity of the D-aminoacyl-tRNAs stems from the suboptimal positioning of the reactive α-amino group, which hinders the nucleophilic attack and blocks the proton shuttle during the peptide bond formation (Figure 5A). As suggested earlier, this suboptimal orientation of the α-amino group of the D-amino acid substrate appears to be caused by the residue U2506 in the PTC, which does not allow such substrate to adopt optimal conformation for the nucleophilic attack (54).

Also, our study might explain previous findings that the use of D-amino acids by the ribosome can be improved through the mutagenesis of the 23S rRNA (32,33). For example, mutations of the ^2447^GAUA^2450^ segment of the *E. coli* 23S rRNA to ^2447^UUGU^2450^ or ^2447^UGGC^2450^ lead to 5-fold improvement of D-phenylalanine or D-methionine incorporation into a reporter protein *in vitro* (32,33). The mutated ^2447^GAUA^2450^ segment of the 23S rRNA is located in the ribosomal A site, with residues G2447 and A2450 being in the direct vicinity of the A-site cleft (Figure 5C). Mutations of the purine nucleotides G2447 and A2450 to smaller pyrimidines should increase the size of the A-site cleft, thereby allowing the D-amino acids to adopt more reactive conformations without clashing of their side chains with the residues of the PTC.

### Implications for the ribosome engineering

Over the past two decades, ribosome engineering produced an array of ribosome variants for applications in basic research and biotechnology (55). Ribosomes with mutated anti-Shine-Dalgarno sequence (56) and with tethered ribosomal subunits (57,58) were constructed to allow the presence of two independent translation systems in a single cell. Hybrids between bacterial and eukaryotic ribosomes were constructed to explore principles of antibiotic specificity (59) or use bacterial ribosomes in eukaryote-derived *in vitro* translation systems (60). Also, engineered ribosomes were produced to decode quadruplet codons (61) or recognize artificial tRNAs (62). Finally, ribosome variants were constructed to improve ribosome compatibility with non-canonical amino acids, such as D-amino acids (32,33) and β-amino acids (63,64).

By showing D-amino acid residue in the ribosomal catalytic center, our structure provides the basis for the rational design of the amino acid binding pocket to improve ribosome compatibility with non-canonical substrates. In this regard, it is important to note that ribosomes with altered A-site cleft have been previously observed in nature. First, the structure of the A-site cleft was shown to vary across species. In eukaryotes, the 80S ribosomes carry C2452U substitution that makes the A-site cleft slightly larger in eukaryotes compared to bacteria and archaea (51). Also, mutations in the A-site cleft or its vicinity are present in mitochondrial ribosomes. In yeast, mitochondrial ribosomes carry G2447A substitution that alters the A-site cleft structure and confers resistance to the A-site targeting antibiotic chloramphenicol, and in mice, mitochondrial ribosomes carry the A2451U substitution that also confers chloramphenicol resistance (65–67). Apart from natural variations of the A site structure, ribosomes with altered A-site cleft were produced in the laboratory by mutagenesis of ribosomal protein L3 and the 23S rRNA residue G2447 (68). Importantly, these mutations are not lethal and only moderately affect the efficiency of protein synthesis (68). Moreover, the impact of the A-site cleft structure on the peptide bond formation was predicted by previous studies employing either theoretical quantum mechanics (69) or molecular dynamics simulations (70). Collectively, previous studies and our structural analyses suggest that the described above ribosome variants with altered A-site cleft might be good candidates for the development of *in vivo* compatible and chirality-promiscuous engineered ribosomes.

In addition to the A-site cleft mutations, a number of alterations have been explored in the ribosomal catalytic center, PTC. Although the majority of the mutations in the PTC are lethal *in vivo*, many of them were studied *in vitro* where they were shown to have either moderate or no effect on protein synthesis. For instance, the use of chemically modified rRNAs for *in vitro* ribosome assembly allowed to introduce highly toxic and lethal mutations or artificial nucleotides into the rRNA (71,72). Using this approach, it was demonstrated that apurinization of the residue U2506 not only preserves catalytic properties of the ribosome but results in a two-fold increase of the yield of *in vitro* protein synthesis (72). Given that this residue prevents the reactive conformation of a D-aminoacyl-tRNA in the A site, its apurinization might improve utilization of the D-amino acids by the ribosome.

The ultimate understanding of the bases for slow incorporation of D-amino acids during ribosomal protein synthesis requires additional structural studies that will illuminate intermediate steps of peptide bond formation and nascent peptide folding in the ribosomal tunnel. Complementary biochemical and microbiological experiments could confirm the predictive power of the structures and create a new generation of synthetic ribosomes for efficient protein synthesis utilizing D-amino acids. While additional research is needed to address these questions, our current study provides the first structural insight into the ancient mechanism by which the ribosome ensures the stereospecific synthesis of natural proteins.

## DATA AVAILABILITY

Coordinates and structure factors were deposited in the RCSB Protein Data Bank with accession code 6N9E for the *T. thermophilus* 70S ribosome in complex with CC-puromycin, mRNA, and P-site tRNA, and 6N9F for the *T. thermophilus* 70S ribosome in complex with ACCA-D-Phe, mRNA, and P-site tRNA.
